# A new species of *Sympistis* Hübner from Sapelo Island, Georgia, USA (Lepidoptera, Noctuidae, Oncocnemidinae)

**DOI:** 10.3897/zookeys.788.26484

**Published:** 2018-10-08

**Authors:** James K. Adams, B. Christian Schmidt

**Affiliations:** 1 Department of Biology, Dalton State College, Dalton, Georgia 30720, USA Dalton State College Dalton United States of America; 2 Canadian National Collection of Insects, Arachnids, and Nematodes, Agriculture and Agri-Food Canada, KW Neatby Bldg., 960 Carling Ave., Ottawa, Ontario K1A 0C6, Canada Canadian National Collection of Insects, Arachnids, and Nematodes Ottawa Canada

**Keywords:** Atlantic coastal fauna, barrier island, dune habitat, *
Sympistis induta
*

## Abstract

A new species of the *Sympistisbadistriga* species-group, *Sympistiseleaner* Adams, **sp. n.** is described from Sapelo Island, a back-barrier island in coastal Georgia, United States of America. Adults and genitalia of *S.eleaner* are illustrated, in addition to adults of similar species in the *Sympistisbadistriga* species-group. The composition of this species-group is discussed.

## Introduction

In 2012, John Hyatt, Dr. Lance Durden, and Dr. Brian Scholtens initiated a study of the moth fauna of Sapelo Island, the fourth largest barrier island in Georgia. Previous to this study, no large barrier island along the southeastern United States coastline has had a fully surveyed Lepidoptera fauna. The proposal for a complete faunal study was discussed with the administrators of the Sapelo Island National Estuarine Research Reserve (SINERR), and with the support of SINERR, the project is now entering its seventh year. Sampling has been carried out in every month several times, and to this point the moth list is over 1000 identified moth species [unpubl. data]. The senior author acted as a taxonomic consultant through the first four years of the Sapelo Island Lepidoptera surveys, and assisted in field work since 2016.

In early May of that year, males of a *Sympistis* Hübner species were collected on Sapelo Island that did not match any of the known species of *Sympistis* from Georgia. Interestingly, this species was not collected in any of the previous years (April–May 2012–2015). Subsequent inquiries to *Sympistis* specialist Jim Troubridge indicated that the specimens possibly represented an unknown species of the *figurata* species-group. Analysis of molecular variation and morphological study revealed that the Sapelo Island *Sympistis* represents a new and likely southeastern endemic species of the *Sympistisbadistriga* species-group, described and illustrated herein as *Sympistiseleaner* sp. n.

## Methods and materials

Wing pattern and genitalia structure terminology follow Troubridge (2008). The structure forming the large, main chamber of the bursa copulatrix was interpreted by Troubridge (2008) to be the appendix bursae, with the small vestigial side chamber representing a highly reduced corpus bursae. Although the vestigial chamber in *Sympistis* is probably correctly interpreted as the corpus bursae (it is variously lost or well-developed in other species, such that positional homologies can be inferred), the appropriate terminology for the main chamber is not clear. The appendix bursae is a blind chamber rather than that which gives rise to ductus seminalis, according to Tuxen (1956). For the sake of consistency with previous terminology in this genus, we follow that of Troubridge (2008), but further research and comparisons are needed to establish homologies and consistent terminology within the Noctuoidea.

Forewing length was measured to the nearest tenth of a millimeter from base to apex, excluding the fringe. Genitalic preparation techniques follow Jaeger (2017). Briefly, abdomens were macerated in 10% KOH solution overnight at room temperature, followed by cursory cleaning, separation of the genitalic capsule, and sequential transfer to 50% EtOH, 70% EtOH, and 95% isopropanol. Vesica and corpus bursae were inflated in 50% EtOH, then transferred to 70% for staining. Two stains (both in ethanol solution) were used, first Chlorazol Black (Fisher Scientific, 112 Colonnade Rd., Ottawa, Ontario) for 10 seconds, then acidified Eosin Y (Fisher Scientific) for 4 + 4 sec in a microwave (Hamilton Beach model #EM720CPN, 700W). Stained tissues were dehydrated in 95% isopropanol before slide-mounting in Euparal. Genitalia were imaged using a Leica DFC450 camera, Leica Application Suite 4.8 and a Leica M205C stereo microscope, and processed in Adobe PhotoShop. Voucher specimens are deposited in the Canadian National Collection of Insects, Arachnids and Nematodes, Ottawa, Ontario, Canada (CNC), and the private collections of the senior author (JKA) and Lance Durden (LDC; 115 Turkey Trail, Statesboro, Georgia, 30458, USA).

The 658 base pair DNA “barcode region” of the mitochondrial cytochrome *c* oxidase subunit 1 (CO1) (“DNA barcode”) was used to assess molecular variation. Legs from dried specimens were submitted to the Barcodes of Life Data Systems (BOLD) at the University of Guelph (Ontario, Canada) where they were analyzed by standard DNA extraction, amplification, and sequencing protocols (Hebert et al. 2003). Barcode sequences were compared to a reference library of nearly all North American *Sympistis* species (Zahiri et al. 2017). Sequence comparisons were made using the Kimura-2-Parameter distance model as implemented on the Barcode of Life Data Systems website (http://www.barcodinglife.org).

## Results

### Sympistis eleaner

Taxon classificationAnimaliaLepidopteraNoctuidae

Adams sp. n.

http://zoobank.org/D1ABF8C9-F690-4F6A-8734-CF051CB7A351

Figs 1 , 2 , 9–11

#### Type material examined.

**Figures 1–8.**
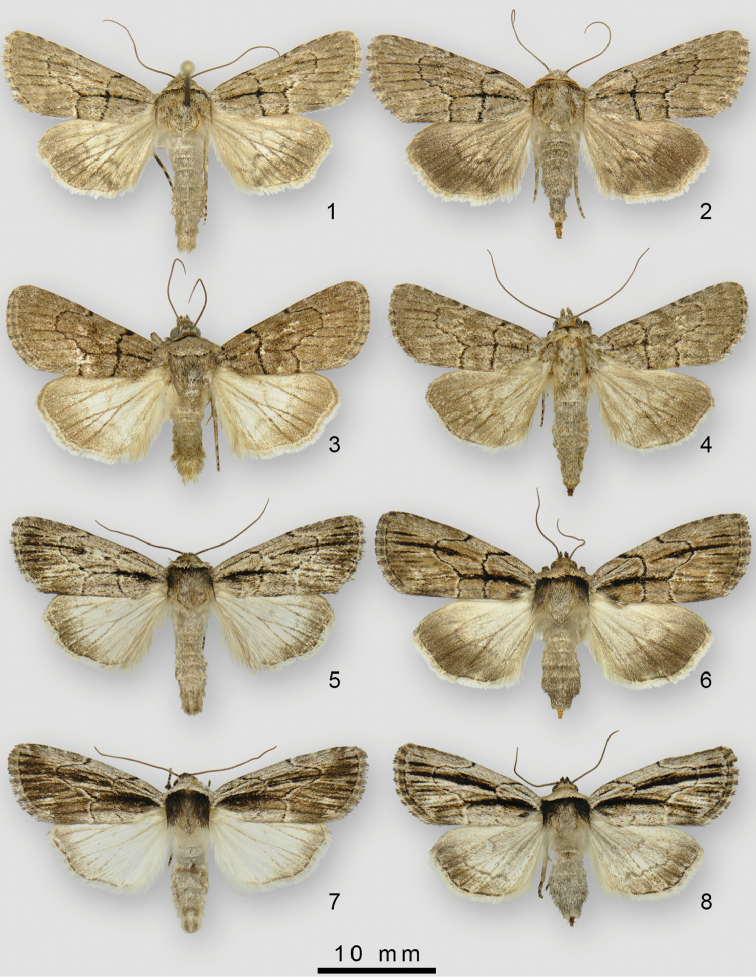
**1***Sympistiseleaner* male (holotype) **2***S.eleaner* female (paratype) **3***S.induta* male **4***S.induta* female **5***S.tenuistriga* male **6***S.tenuistriga* female **7***S.badistriga* male **8***S.badistriga* female.

All specimens from the US. **Holotype** male (Fig. 1). Georgia: McIntosh Co., Sapelo Island, Dune habitat, just S of Beach Rd., light trap, 31°23'26.5"N, 81°15'54.5"W, May 6–7, 2016, James K. Adams, DNA voucher # CNCLEP00119937 [CNC]. **Paratypes** (3 males, 2 females). **Georgia**: Same location as holotype, May 7–8, 2016 (2 males); McIntosh Co., Sapelo Island, Greenhouses, 21–22 April 2017, MV light sheet, L. A. Durden & T. Matson, (1 female); McIntosh Co., Sapelo Island, nr. UGA dorms, 31°23'54"N, 81°16'51"W, 9 May 2017, B. Scholtens (1 female; Fig. 2); McIntosh Co., Sapelo Island, “Short Cut” Rd., 31°24'36"N, 81°17'3"W, 9–10 May 2017, light trap, J. Adams and B. Scholtens (1 male) [CNC, JKA, LDC].

#### Diagnosis.

*Sympistiseleaner* (Figs 1, 2) is most similar in appearance to *S.induta* (Harvey) (Figs 3, 4) but the distribution of the two do not overlap, with *S.induta* restricted to Texas. *Sympistiseleaner* is not likely to be confused with any other species *Sympistis* besides *S.induta*. There is a set of rather subtle, but distinct, pattern elements that distinguish the two species: *S.eleaner* has an overall streakier appearance, especially in the male hindwing; the forewing antemedial line takes a distinctive 110° turn inward at the costa that is more irregular in *S.induta*; there is a lighter patch near the anal angle of the hindwing (between Cu2 and A2) that is not present in *S.induta*; the male of *S.induta* has a much “cleaner” hindwing with a less irregular PM line; the female forewing is broader than that of the male in *S.eleaner*, but female *induta* have narrower forewings than the males.

**Figures 9–11.**
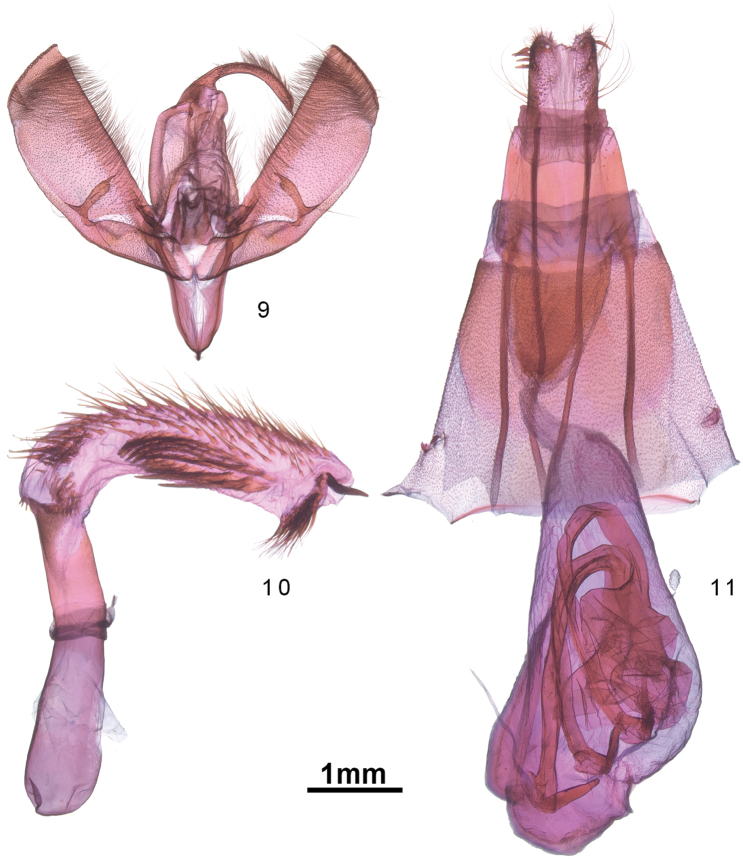
**9***S.eleaner* male valve **10** Phallus **11** female bursa copulatrix

The male genitalia of *S.eleaner* (Figs 9, 10) differ from those of *S.induta* and other species in the *badistriga* species-group by the shorter, broader valve that has a nearly linear costal margin. In females, the combination of a pear-shaped corpus bursae with a relatively short ductus bursae is unique.

**Description.** Forewing pattern typical of the *Sympistisbadistriga* species-group, beige, dark markings limited to sinuous antemedial and postmedial lines. Male and female similar, females slightly larger and darker, especially the distal hindwing (Figs 1, 2). ***Head*.** Labial palpus, frons, head vertex scales light gray beige and scattered dark brown; female darker with more scattered black scales; antenna simple, unscaled; eye without interfacetal setae; labial palpus with heavily scaled basal and second segments, second segment mottled prominently dark brown; third segment very short, stout. ***Thorax*.** Vestiture grayish beige, concolourous with abdomen and forewing ground; black prothoracic collar pronounced in male, incomplete medially in female; collar with posterior linear extension onto mesonotum in male; leading edge of mesonotum (directly behind collar) with fringe of hair-like, dark brown scales, making collar appear more pronounced. Legs with distal end of femur with black scaling (at “knee”); proximal 2/3 of tarsomeres 1 and 2 on all legs black, proximal ½ on remaining tarsomeres black; paired tibial spurs, scaled black, on meso- and metathoracic legs; foretibial spur present but concolorous with rest of thorax; stout apical spur of foretibia typical of many *Sympistis* absent. ***Forewing*.** Male forewing length 13.7–14.0 mm, female 14.9 mm. Dorsal forewing ground color grayish beige with dusting of black scales, most concentrated along veins; basal dash extending to postmedial line along posterior edge of discal cell; antemedial line with distinct bend at costa (approx. 110°); posteriorly angled slightly outward to anal edge; postmedial line with typical *Sympistis*-bulge from end of basal to costa; costa distad of postmedial line with alternating light beige spots at radial veins and dark gray dashes between veins; fringe with alternating beige at veins and dark grayish tan between; distal half of ventral forewing cream beige to cream (especially in anal area); scattered dark brown scales, denser toward both costa and apex, costa and fringe checkered as above; faint postmedial line short, from radial veins to M2; abundant tan hair-like scales in discal cell. ***Hindwing*.** Dorsum cream to cream beige basally and along anal; outer fringe scales two-toned, beige basally, cream terminally (no checkering); postmedial line sinuous and discontinuous, separate from outer band in male, fused in female; gray outer band also discontinuous, darkest along veins, lightest at anal angle between Cu and A2. Venter basal 2/3 cream beige to cream; outer band darker distally to smooth curving unbroken PM line (from costa to Cu1); small patch of dark brown scales demarcate small discal spot on both fore- and hindwing ventrum. ***Abdomen*.** Vestiture grayish beige; female with more extensive dark scaling; black scales concentrated in two ventrolateral dark lines running length of abdomen; male with basal abdominal coremata, levers and associated pocket well-developed; Stobbe’s gland well-developed; female abdomen unmodified. ***Male genitalia*.** Valve elongate with distal 2/3 somewhat rounded-quadrate, with costal edge nearly linear and distal margin only slightly convex; clasper slightly bulbous with claw-like apex, curving mesad slightly; corona a single row of robust but deciduous spine-like setae; juxta poorly differentiated; uncus curved moderately ventrad, tapering rather abruptly at apex, with small, nearly caudad-directed apical spine; saccus shaped as somewhat convex/curved V; phallus cylindrical and slightly decurved ventrad; distal half slightly narrower than basal portion, length 5.3 × that of diameter; vesica produced at 90° right dorso-laterad, with four spine fields and a terminal cornutus (Fig. 10). ***Female genitalia***. Papillae anales slender with somewhat rounded distal margin; distally with short sparse setae and a row of subterminal, lateral hook-like spines forming a corona; posterior apophysis long and slender, 3 × length of sclerotized portion of A8; posterior apophysis 2.5 × length of sclerotized portion of A8; lamella antevaginalis weakly sclerotized and unmodified; ostium forming a rounded cone, slightly longer than wide; appendix bursae pear shaped, with ductus seminalis originating at anterior end and directed caudad; corpus bursae reduced to a small, polyp-like vesicle attached to right side of main bursa chamber.

#### Etymology.

The species is named in honor of the mother of JKA, Eleaner Ruth Adams. She continuously encouraged JKA in studying Lepidoptera from a very young age and participated with JKA in many moth outings during her life.

#### Biology and Distribution.

Nothing is known about the early stages of *S.eleaner*. Larval hosts for related species in the *badistriga* species-group are known or thought to be primarily species in the honeysuckle family (Caprifoliaceae); *S.badistriga* (Grote) feeds on *Lonicera* Linnaeus and *S.stabilis* (Smith) feeds on *Symphoricarpos* Duhamel, respectively (Crumb 1956). At least three species of Caprifoliaceae are known to occur on the island: *Sambucussimpsonii* Rehder, *Lonicerajaponica* Thunberg, and *L.sempervirens* Linnaeus (Chalmers 1997). The recorded flight time for the species is from April 21 to May 10; it likely flies a bit earlier and/or later than this date range depending on the year. *Sympistiseleaner* is probably univoltine like other members of the species group, and since it has not been collected during any other season despite intensive sampling, although the fact that it took five years to find the moth in the first place does not rule out the possibility of later-flying generations.

The type locality is a large, stabilized dune 0.4 km from the Atlantic shoreline. It is vegetated sparsely (most notably by *Cenchrustribuloides* Linnaeus (Poaceae) and short *Smilax* Linnaeus (Smilacaceae)) and surrounded by southern red cedar (*Juniperussilicicola* (Small) Bailey, Cupressaceae), various pines (*Pinus* Linnaeus, Pinaceae) and scrub oaks (*Quercus* Linnaeus, Fagaceae). The known distribution of *Sympistiseleaner* is currently defined by the type series. These localities are within a 2.8 km section along the Autobahn/Beach Road on the south side of Sapelo Island. It has been found in dune, grassy (surrounded by forest at the greenhouse) and forested habitats. It could potentially occur on other barrier islands along the southeastern United States coast, but extensive surveys may need to be done to find it considering the species is rarely and only recently collected on Sapelo Island.

#### Molecular variation.

DNA barcode sequence (voucher # CNCLEP11937) of the holotype male formed a unique Barcode Index Number (BOLD:ADG0355), differing by a minimum of 5% from all other North American *Sympistis* species, but consistently clustering with species of the *badistriga* and *infixa* species-groups.

#### Discussion.

*Sympistis* is the second-largest genus of noctuids in North America, with 178 species (Pohl et al. 2016). It is most diverse in xeric habitats of the western United States. Just four species have been recorded previously from Georgia: *S.badistriga*, *S.infixa* (Walker), *S.perscripta* (Guenée), and *S.chionanthi* (Smith). The genus was reviewed by Troubridge (2008), who described many new species (all western), and synonymized eight genera under a revised concept of *Sympistis*. Troubridge (2008) recognized 19 species-groups, defined largely by genitalic morphology, that incorporate about half of the known species.

The forewing traits of *S.eleaner*, with a simple, streaky pattern, dark basal dash and evenly sinuate antemedial and postmedial lines, are shared with members of the *infixa* and *figurata* species-groups. Although these groups are externally similar, they differ significantly in morphology. A subgroup of the *S.badistriga* species-group (Troubridge 2008) comprised of *S.badistriga*, *S.apposita* (Barnes and McDunnough), *S.stabilis*, *S.induta*, *S.rayata* (Smith) and *S.tenuistriga* (McDunnough) is defined by absence of the stout foretibial spine, presence of a tiny, vestigial corpus bursae, and the origin of the ductus seminalis from the anterior appendix bursae. *Sympistiseleaner* exhibits all of these synapomorphies, placing it securely within the *S.badistriga* species-group. However, neither its barcode nor morphological features suggest a close relationship to any particular species, seemingly representing a distinct and likely relict eastern member within the group with a long, separate evolutionary history. This underscores the need for a phylogenetic framework for this very large, morphologically and ecologically diverse genus.

## Supplementary Material

XML Treatment for Sympistis eleaner
